# Development of a HTRF® Kinase Assay for Determination of Syk Activity

**DOI:** 10.2174/1875397300801010020

**Published:** 2008-02-25

**Authors:** Christopher Harbert, Jeannette Marshall, Sharon Soh, Krista Steger

**Affiliations:** Cisbio US Inc., 12 Deangelo Dr., Bedford MA 01730, USA

## Abstract

Regulation of protein phosphorylation is a primary cellular signaling mechanism. Many cellular responses to internal and external events are mitigated by protein kinase signaling cascades. Dysfunction of protein kinase activity has been linked to a variety of human pathologies, in the areas of cancer, inflammation, metabolism, cell cycle, apoptosis, as well as cardiovascular, neurodegenerative and autoimmune diseases [1-3]. As such, there is an important need for protein kinase activity detection methodologies for researchers engaged in Drug Discovery. A number of different technologies have been employed for the measurement of protein kinase activity, including radioactive methods, luminescent methods, and fluorescent methods. More recently, Homogeneous Time Resolved Fluorescence technology (HTRF^®^), based on the principle of time-resolved fluorescent resonance energy transfer (TR-FRET), has been developed and applied for the measurement of protein kinase activity *in vitro*. This technology note describes the development of an HTRF^®^ assay for detection of Syk enzyme activity in a format consistent with the requirements of High-Throughput Screening (HTS) campaigns currently used in drug discovery.

## INTRODUCTION

Kinases are one of the most interesting target families in drug discovery [4]. Mis-regulation of protein kinase activity has been implicated in numerous conditions, including cancers, inflammatory, and metabolic diseases [5,6]. The protein kinase family of enzymes contains more than 500 members, and is comprised of two sub-families, protein tyrosine (TK) and protein serine-threonine (STK), which mediate a wide spectrum of actions [7].

A number of detection methodologies have been applied to protein kinase activity measurement. Radioactive methodologies, such as those utilizing scintillant-containing bead or plate surfaces, are generally considered the gold-standard for referencing kinase activity statistics, such as K_m_, k_cat_, and lead compound statistics, such as IC_50_. However, non-isotopic, homogeneous methods are increasing preferred for HTS settings [8]. Luminescent-based technologies, including Enzyme Fragment Complementation assays, ELISA systems, and Amplified Luminescent Proximity Homogeneous Assay technology exhibit certain limitations due to the destructive nature of the assay readout. In these technologies, detection is a one-time-only event, and it is not possible to repeat readings of assay plates. Fluorescent-based detection offers greater flexibility, since multiple readings of the same assay plate are possible. Prompt fluorescence technologies typically exhibit high levels of background signals, limiting the sensitivity of these methods. In addition, the use of a single fluorophore may increase the likelihood of compound interference.

The HTRF^®^ technology is used by leading drug discovery researchers to screen for tyrosine and serine/threonine kinase inhibitors which provides a simple and robust assay platform for compound screening. HTRF^®^ is a versatile TR-FRET technology developed for detecting molecular interactions between biomolecules, and has been successfully applied to protein kinase activity bioassays with purified enzymes, as well as with cellular lysates and membrane preparations [9-11]. All HTRF^®^ kinase assays follow the same general format, which includes a phosphospecific antibody conjugated to Eu^3+^ cryptate (donor fluorophore), and an anti-tag or affinity molecule conjugated to XL665 or d2 (acceptor fluorophore) which recognizes the substrate, irrespective of its phosphorylation state. The time-resolved characteristic of HTRF^®^ technology allows for the removal of nearly all environmental and compound effects. The use of a FRET-based readout has an additional advantage of specificity for the intended biological moiety under investigation. HTRF^®^ reagents are unaltered in the presence of divalent cations, such as Mg^2+^ or Mn^2+^, and tolerate virtually any concentration of EDTA or ATP. HTRF^®^ chemistry is also able to withstand high concentrations of DMSO and other standard laboratory solvents. These features offer researchers involved in protein kinase activity monitoring a robust, flexible platform with which to perform their studies.

Detection of phosphorylation events have been validated using HTRF^®^ on small peptides as well as large protein substrates [12]. The use of specific, physiologic substrates is generally preferred; however, production and purification of these substrates can be challenging. For this reason, many researchers look to small peptide substrates to facilitate protein kinase activity screening. Universal substrates are also an attractive concept, given the dramatic reduction in lead time for assay development that can be achieved using this approach.

For tyrosine kinase assays, two universal substrates, poly-Glu:Tyr (pGT) and poly-Glu:Ala:Tyr (pGAT) are available in the HTRF assay format. These two large peptide substrates (MW range: 25kDa & 44kDa) are useful tools for measuring tyrosine kinase activity, and yet there has been some concern among enzymologists about the kinetics of such a phosphorylation event. To address these concerns, a new HTRF^®^ KinEASE^™^ kit was developed. It uses a unique biotinylated peptide substrate containing a single tyrosine phosphorylation site, which when phosphorylated is recognized by a monoclonal anti-phospho-tyrosine antibody, labeled with Eu^3+^cryptate. The detection is completed by the addition of streptavidin-XL665. This combination of substrate and detection reagents has been validated on 59 unique tyrosine kinases, including both cytosolic and receptor-like TK’s. The HTRF^®^ KinEASE^™^ assay platform is compatible with any ATP concentration, is easily miniaturized and has been successfully used for protein kinase selectivity studies as well as high-throughput screening campaigns.

In this study, tyrosine kinase Syk was used to compare the different assay formats available in HTRF^®^ readout.

### Syk Enzyme Role in Physiology

Spleen tyrosine kinase (Syk) is a non-receptor tyrosine kinase which has been linked to a variety of human signaling cascades. It is required for the transduction of immunoreceptor signals following antigen presentation. Syk is involved in B-cell receptor signaling, IgE receptor signaling in mast cells, and has been demonstrated to be essential for lymphocyte development in knock-out studies [13]. Because of its role, Syk has become a drug target for potential therapies in allergy and autoimmunity disorders [14-17]. Besides its prominent role in the immune system, Syk has also been reported to assist in tumor suppression in breast cancer, where it has key functionality with regard to restricting cellular motility through its interaction with the actin and tubulin cytoskeleton [18, 19].

## MATERIALS AND METHODS

### Buffers

Enzyme, substrates and ATP are prepared in the enzymatic buffer, which consists of 50 mM HEPES pH 7.0, 0.02% NaN3, 0.01% BSA 0.1mM Na2VO3, 5 mM MgCl2, 5 mM MgCl2 and 1mM DTT. Detection reagents are prepared in detection buffer containing 50 mM HEPES pH 7.0, 0.1% BSA, 0.8 M KF and 20 mM EDTA.

### Tyrosine Kinase Assays

There are two main steps in the kinase assay: kinase reaction and detection of the phosphorylated product by HTRF reagents. The kinase reaction was started by addition of ATP to a mixture containing the enzyme and substrates. After 10 minutes or 30 minutes incubation at room temperature, the enzyme reaction was stopped by EDTA-containing buffer, which also contained europium-conjugated anti-phosphoresidue antibody and streptavidin-XL665(SA-XL665) to allow for detection of the phosphorylated peptide product. Following 1 hour incubation at room temperature fluorescence was measured with excitation of 337 nm and dual emission of 665 and 620 nm on the RubyStar microplate reader (BMG Lab Technologies). Signal was expressed in terms of HTRF ratio (fluorescence intensity @ 665 nm/fluorescence intensity @ 620nm x 10,000).

The detailed assay protocol for SyK kinase assay is summarized in Table **2**. The assay can be run in any plate format (96-, 384-, 1536-, 3456-well) by resizing each reagent volume proportionally. The experiments described here were performed in a final assay volume of 20 μl, with n=3 replicates for each condition.

Details of experimental steps for Syk activity assay in 384-lv plate format are listed in Table **2**.

## RESULTS AND DISCUSSION

Detection of phosphorylation events have been validated using HTRF^®^ on small peptides as well as large protein substrates [12]. The use of specific, physiologic substrates is generally preferred; however, production and purification of these substrates can be challenging. For this reason, many researchers look to small peptide substrates to facilitate protein kinase activity screening. Universal substrates are also an attractive concept, given the dramatic reduction in lead time for assay development that can be achieved using this approach.

For tyrosine kinase assays, two universal substrates, poly-Glu:Tyr (pGT) and poly-Glu:Ala:Tyr (pGAT) are available in the HTRF assay format. These two large peptide substrates (MW range: 25kDa & 44kDa) - activity, and yet there has been some concern among enzymologists about the kinetics of such a phosphorylation event. To address these concerns, a new HTRF^®^ KinEASE^™^ kit was developed recently. It uses a unique biotinylated peptide substrate containing a single tyrosine phosphorylation site, which when phosphorylated is recognized by a monoclonal anti-phospho-tyrosine antibody, labeled with Eu^3+ ^cryptate. The detection is completed by the addition of streptavidin-XL665. This combination of substrate and detection reagents has been validated on 59 unique tyrosine kinases, including both cytosolic and receptor-like TK’s. The HTRF^®^ KinEASE^™^ assay platform is compatible with any ATP concentration, is easily miniaturized and has been successfully used for protein kinase selectivity studies as well as high-throughput screening campaigns.

The first step in the development of an HTRF^®^ kinase assay is to determine a rough working range of enzyme and substrate. This is normally accomplished by running a matrix of enzyme titration versus substrate titration, with high levels of ATP (~100μM). Titrations of Syk enzyme were run at various concentrations of poly-GT-biotin, poly-GAT-biotin, or TK substrate-biotin. ATP was held constant at 100µM. The enzymatic reaction was allowed to proceed for either 10 minutes or 30 minutes at room temperature. SA-XL665 was kept in constant molar proportion (1:8) to the biotinylated substrate in all cases. This constant proportion of streptavidin to biotin is necessary to avoid masking signals at the top and bottom titration points.

### HTRF^®^ Syk Assay with Poly-(GT)-Biotin and Poly-(GAT)-Biotin Substrates

First, we established kinase activity levels at various enzyme and substrate concentrations. ATP was held constant at 100μM, to avoid limiting reaction progress due to ATP consumption. Syk enzyme showed higher activity with poly-GT-biotin than with poly-GAT-biotin (data not shown). With poly-GT-biotin, MAb PT66-K produced better signal detection than MAb PY20-K (data not shown). Reaction times of 10 minutes produced less signal than reaction times of 30 minutes in all cases, as expected (data not shown). Fig. (**2**) shows titration of poly-GT-biotin using various concentrations of Syk enzyme, and detection with MAb PT66-K and SA-XL665.

The assay system showed a signal increase which was both Syk-dependant and poly-GT-biotin dependant, indicating signal obtained is reflective of substrate phosphorylation. Under these conditions, 6.8nM Syk and 0.1μM substrate showed the best compromise between signal:noise ratio and linearity of response. This data highlights that the limiting reagent in this reaction is Syk enzyme, as evidenced by the significant drop off in activity with decreasing enzyme concentration.

### HTRF^®^ Syk Assay with KinEASE TK Substrate-Biotin

The Syk enzyme activity was much more efficient with the KinEASE TK substrate. Fig. (**3**) shows a comparison of all tested substrates at an enzymatic concentration of 1μM substrate.

Syk enzyme showed dramatically higher signal output with TK substrate-biotin than either poly-GT-biotin or poly-GAT-biotin. There are several explanations for this result. First, the KinEASE TK substrate may be a more functional substrate for Syk than poly-GT- or poly-GAT-biotin. The KinEASE TK substrate was developed for its ability to elicit high levels of activity from a broad panel of tyrosine kinases. Second, the affinity of KinEASE TK MAb for its substrate may be higher than that of PY20 or PT66 for either poly-GT-biotin or poly-GAT-biotin. Higher affinity interactions increase the probability of higher signal generation, since it is more likely to find partners in a bound step during measurement. Finally, the KinEASE TK substrate has a signal phosphorylation site, while the other substrates have 20 or more potential phosphorylation sites (determined by sequence and molecular weight). As the anti-phospho MAb is generally limiting for signal output, it is possible that some fraction of antibody may bind to phosphorylated tyrosines on poly-GT-biotin or poly-GAT-biotin which, because of their orientation and distance, do not allow FRET signaling to occur. This would limit the maximum signal achievable by the system. It is important to note, however, that while the signal output was greatly increased with the use of KinEASE TK substrate, significant signal output was achieved with all three substrates.

From this experiment, the optimal concentration would be 1.36nM Syk enzyme with the 1µM KinEASE TK substrate. This condition represents excellent signal:noise performance, while maintaining good linearity of the reaction, as evidenced by the shape of the enzyme titration curve.

As the performance of KinEASE TK substrate with Syk was so dramatic at 1μM, we then attempted to see whether acceptable assay performance could be obtained at lower substrate concentrations. Fig. (**4**) shows the results of Syk titration at 0.1μM and 0.01µM substrate.

From this experiment, the optimal concentration would be 1.36nM Syk enzyme with the 0.1µM KinEASE TK substrate. This condition represents excellent signal:noise performance, while maintaining good linearity of the reaction, as evidenced by the shape of the enzyme titration curve. While maximum signal:noise ratio is lower in this experiment than in the previous, there are multiple advantages to utilizing 10-fold less substrate. By using lower concentrations of substrate during the reaction step, the threshold for non-ATP competitive inhibitor potency is similarly lowered. In other words, a broader spectrum of non-ATP competitive inhibitor potencies can be detected. Additionally, since the consumption of SA-XL665 is tied proportionally to the consumption of substrate, reducing substrate concentrations during the enzymatic step simultaneously reduces assay cost per well.

The optimal condition as determined from the three experiments depends greatly on the specific needs of the researcher. If non-ATP competitive inhibitors are not desired, high concentration of substrate can be used, and enzyme consumption can be reduced. This is also a good option if enzyme is scarce or difficult to produce. If enzyme is abundant, or if non-ATP competitive inhibitors are strongly desired, substrate concentration can be minimized while maintaining acceptable signal to noise levels for screening campaigns.

### Determination of Syk Enzyme Kinetics

Following enzyme and substrate range determination, optimization of two further parameters is generally required before embarking on compound screening. K_m_ (apparent) determination for ATP and substrate gives the researcher an understanding of the relative potency required for inhibitors to demonstrate activity under selected assay conditions. In this study we performed a K_m_ analysis for the KinEASE TK substrate, using non-limiting ATP (100µM). Results are shown in Fig. (**5**).

The KinEASE TK substrate showed apparent K_m_ values of approximately 0.1µM with R-squared values >0.98 in all cases. Researchers in search of non-ATP competitive inhibitors generally would screen using a substrate concentration at or below apparent K_m_. Researchers interested in only ATP-competitive inhibitors generally would screen using a substrate concentration several fold (5-fold or more) about apparent K_m_.

### IC_50_ of Known Inhibitors

As validation of the Syk/KinEASE TK assay system, we ran a titration of staurosporine, a known ATP-competitive inhibitor, using KinEASE TK substrate at 200nM, and ATP at 10µM. Results are shown in Fig. (**6**).

Staurosporine showed expected inhibition of Syk activity, with an IC_50_ value of 9.9nM, and an R-squared value above 0.97 for curve fit. This value is consistent with other reported values from the literature for staurosporine inhibition of Syk [20-22].

## CONCLUSIONS

To further meet the needs of scientists involved in protein kinase research, the HTRF^®^ KinEASE^™^ TK Kit has been developed as a unique and powerful tool for the screening of tyrosine kinases. With one biotinylated substrate, containing a single phosphorylation site, enzymatic activity can be measured for at least 59 unique tyrosine kinases with a single product. The performance of the unique TK substrate-biotin, as compared to other universal tyrosine kinase substrates, provides researchers vast flexibility in their assay design without sacrificing assay performance. Due to its single phosphorylation site, kinetic determinations such as K_m_ apparent are easily investigated.

Because of KinEASE^™^ TK’s impressive signal to noise performance, enzyme and/or substrate concentrations can be minimized or maximized in order to suit the needs of each individual researcher. With enzymes at times being costly or difficult to produce, KinEASE^™^ TK allows for a dramatic reduction in enzyme consumption while maintaining performance requirements for screening. With its large dynamic range of functional substrate concentrations, and its applicability to any ATP concentration, KinEASE^™^ TK allows scientists the opportunity to customize screening conditions to target the discovery of ATP-competitive inhibitors, ATP non-competitive inhibitors, or both. Additional information for HTRF^®^ kinase assays and other related reagents can be found on the web at www.HTRF.com.

## Figures and Tables

**Fig. (1). A typical HTRF® protein kinase assay setup.**
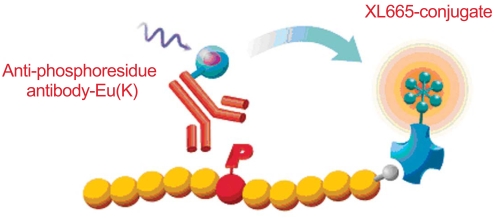
HTRF^®^ protein kinase assays are characterized by the use of specific anti-phosphoresidue antibodies, generic affinity tags on the substrate molecule of interest, and specific anti-tag reagents for substrate catching.

**Fig (2). Titration of poly-GT-biotin vs. Syk enzyme.**
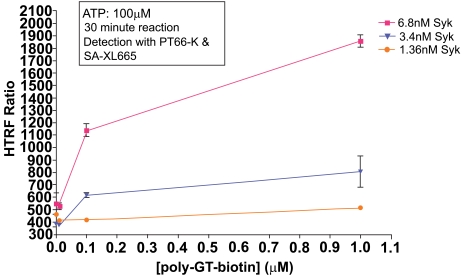
Various concentrations of Syk enzyme were run using different concentrations of poly-GT-biotin substrate. The optimal condition (6.8nM Syk, 0.1µM substrate) produced a signal to background ratio of 8.1.

**Fig. (3). Titration of Syk enzyme at 1µM substrate.**
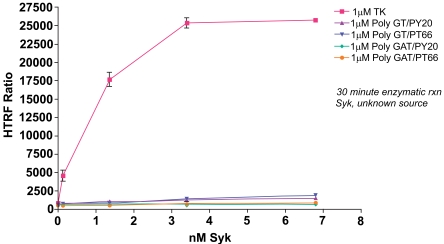
Various concentrations of Syk enzyme were run using different substrates at a concentration of 1µM. The TK substrate-biotin showed the greatest performance, with the optimal condition (1.36nM Syk, 1µM TK substrate-biotin) producing a signal to background ratio of 43.9.

**Fig. (4). Titration of Syk enzyme at 0.1µM substrate.**
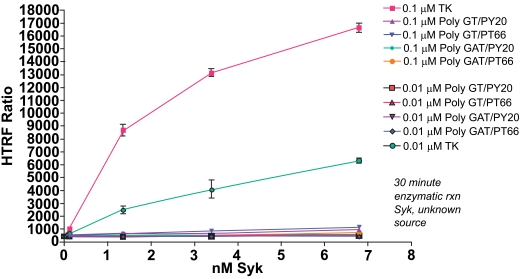
Various concentrations of Syk enzyme were run using different substrates at a concentration of 0.1µM or 0.01 µM. The TK substrate-biotin showed the greatest performance under either concentration, with the optimal condition (1.36nM Syk, 0.1µM TK substrate-biotin) producing a signal to background ratio of 21.5.

**Fig. (5). Km determination for KinEASE TK substrate with Syk enzyme.**
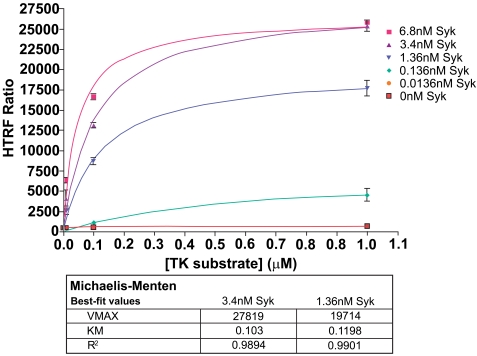
Various concentrations of Syk enzyme were run using different concentrations of TK substrate-biotin. Michaelis-Menten analysis of results fluorescence yielded a K_m_ apparent value of 0.1µM TK substrate-biotin. The signal to noise ratio at this concentration of substrate (3.4nM Syk) was 30.7.

**Fig (6). Staurosporine inhibition of Syk activity.**
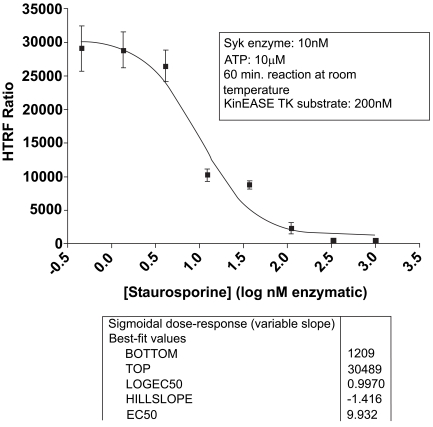
Various concentrations of the ATP-competitive inhibitor Staurosporine were incubated with Syk enzyme, prior to initiation of the kinase reaction. Analysis of the resulting fluorescence yielded an IC_50_ concentration of 9.9nM. This value is consistent with previous published results.

**Table 1:** Materials. Major Materials used in the Assay are Listed in Table **1**.

Name (Source)	Description	Catalog Number	Note
ATP (Sigma-Aldrich)		A7699	
Poly-GT-biotin (Cisbio)	Tyrosine kinase substrate	61GT0BLD	44KDa; Sequence: (GT)(4:1)
Poly-GAT-biotin (Cisbio)		61GATBLB	25KDa, Sequence: (GAT)(6:3:1)
TK substrate-biotin (Cisbio)		62TK0PEB	1.5KDa;*
PT66-K (Cisbio)	Europium conjugated antibody specific for phosphorylated tyrosine residue	61Y20KLB	
PY20-K (Cisbio)		61Y20KLB	
TK antibody (Cisbio)		62TK0PEB	
Streptavidin-XL665 (Cisbio)	Detection reagent	610SAXLB	
Staurosporine (Sigma-Aldrich)	ATP-competitive inhibitor	S4400	
384-lv plate (Greiner)	Black polystyrene	784067	
RubyStar^®^ (BMG)	Microplate reader	N/A	
Syk enzyme	Tyrosine kinase	N/A	73kDa; specific activity = 96pmol/µg x min

* sequence provided by Cisbio to KinEASE TK users by Cisbio upon request.

**Table 2:** HTRF® Kinase Assay Protocol

Step	Parameter	Value	Description
1	Compound	0.5 µL	DMSO 50%, HTRF^®^ Enzymatic buffer 1x
2	Enzyme	5.5 µL	Dilution in HTRF^®^ Enzymatic buffer 1x
3	Incubation	15min	Room temperature, covered with plate seal
4	Substrate	2 µL	Dilution in HTRF^®^ Enzymatic buffer 1x
5	ATP	2 µL	Dilution in HTRF^®^ Enzymatic buffer 1x
6	Incubation	10 or 30min	Room temperature, covered with plate seal
7	Reagent	10 µL	Europium Anti-P-MAb, SA-XL665 in HTRF^®^ Detection buffer
8	Incubation	60 min	Room temperature
9	Detection	HTRF^®^ mode	Rubystar plate reader

Note: All experiments in this study were performed and completed in two working days.

## References

[1] Levitzki A, Gazit A (1995). Tyrosine kinase inhibition: an approach to drug development. Science.

[2] Levitzki A (1994). Signal-transduction therapy. A novel approach to disease management. Eur J Biochem.

[3] Wong WS (2005). Inhibitors of the tyrosine inase signaling cascade for asthma. Curr Opin Pharmacol.

[4] Cohen P (2002). Protein kinases--the major drug targets of the twenty-first century?. Nat Rev Drug Discov.

[5] Dancey J, Sausville EA (2003). Issues and progress with protein kinase inhibitors for cancer treatment. Nat Rev Drug Discov.

[6] Adams JL, Badger AM, Kumar S, Lee JC (2001). p38 MAP kinase: molecular target for the inhibition of pro-inflammatory cytokines. Prog Med Chem.

[7] Manning G, Whyte DB, Martinez R, Hunter T, Sudarsanam S (2002). The protein kinase complement of the human genome. Science.

[8] Smith C O'Donnell J The Process of New Drug Discovery and Development 2006 2nd Boca Raton CRC Press

[9] Bader B, Klotz M, Zopf D, Glienke J, Martinez S Receptor Tyrosine Kinase Screening - Closer To Physiology.

[10] Park YW, Cummings RT, Wu L, Zheng S, Cameron PM, Woods A, Zaller DM, Marcy AI, Hermes JD (1999). Homogeneous Proximity Tyrosine Kinase Assays: Scintillation Proximity Assay versus Homogeneous Time-Resolved Fluorescence. Anal Biochem.

[11] Jia Y, Quinn CM, Clabbers A, Talanian R, Xu Y, Wishart N, Allen H (2006). Comparative analysis of various *in vitro* COT kinase assay formats and their applications in inhibitor identification and characterization. Anal Biochem.

[12] Chambon M Screening and profiling for kinases: development of a unique and versatile platform. Miptec 2005, Basel, Switzerland.

[13] Turner M, Schweighoffer E, Colucci F, Di Santo JP, Tybulewicz VL (2000). Tyrosine kinase SYK: essential functions for immunoreceptor signalling. Immunol Today.

[14] Kyttaris VC, Tsokos GC (2007). Syk kinase as a treatment target for therapy in autoimmune diseases. Clin Immunol.

[15] Wong WS (2005). Inhibitors of the tyrosine kinase signaling cascade for asthma. Curr Opin Pharmacol.

[16] Wong BR, Grossbard EB, Payan DG, Masuda ES (2004). Targeting Syk as a treatment for allergic and autoimmune disorders. Expert Opin Investig Drugs.

[17] Yamamoto N, Hasegawa H, Seki H, Ziegelbauer K, Yasuda T (2003). Development of a high-throughput fluoroimmunoassay for Syk kinase and Syk kinase inhibitors. Anal Biochem.

[18] Navara CS (2005). The spleen tyrosine kianse (Syk) in human disease, implications for design of tyrosine kinase inhibitor based therapy. Curr Pharm Des.

[19] Coopman PJ, Mueller SC (2006). The Syk tyrosine kinase: a new negative regulator in tumor growth and progression. Cancer Lett.

[20] Yamamoto N, Hasegawa H, Seki H, Ziegelbauer K, Yasuda T (2003). Development of a high-throughput fluoroimmunoassay for Syk kinase and Syk kinase inhibitors. Anal Biochem.

[21] Zhu X, Kim JL, Newcomb JR, Rose PE, Stover DR, Toledo LM, Zhao H, Morgenstern KA (1999). Structural analysis of the lymphocyte-specific kinase Lck in complex with non-selective and Src family selective kinase inhibitors. Structure.

[22] Atwell S, Adams JM, Badger J, Buchanan MD, Feil IK, Froning KJ, Gao X, Hendle J, Keegan K, Leon BC, Müller-Dieckmann HJ, Nienaber VL, Noland BW, Post K, Rajashankar KR, Ramos A, Russell M, Burley SK, Buchanan SG (2004). A Novel Mode of Gleevec Binding Is Revealed by the Structure of Spleen Tyrosine Kinase. J Biol Chem.

